# Supplementary material for: Immunotherapy Enhancement by Targeting Extracellular Tumor pH in Triple-Negative Breast Cancer Mouse Model
Source: Cancers (Basel). 2023 Oct 11;15(20):4931. doi: 10.3390/cancers15204931 (PMC10605606; doi:10.3390/cancers15204931)
Supplement: Supplementary file 1 [file cancers-15-04931-s001.zip › cancers-2603626-supplementary.pdf]

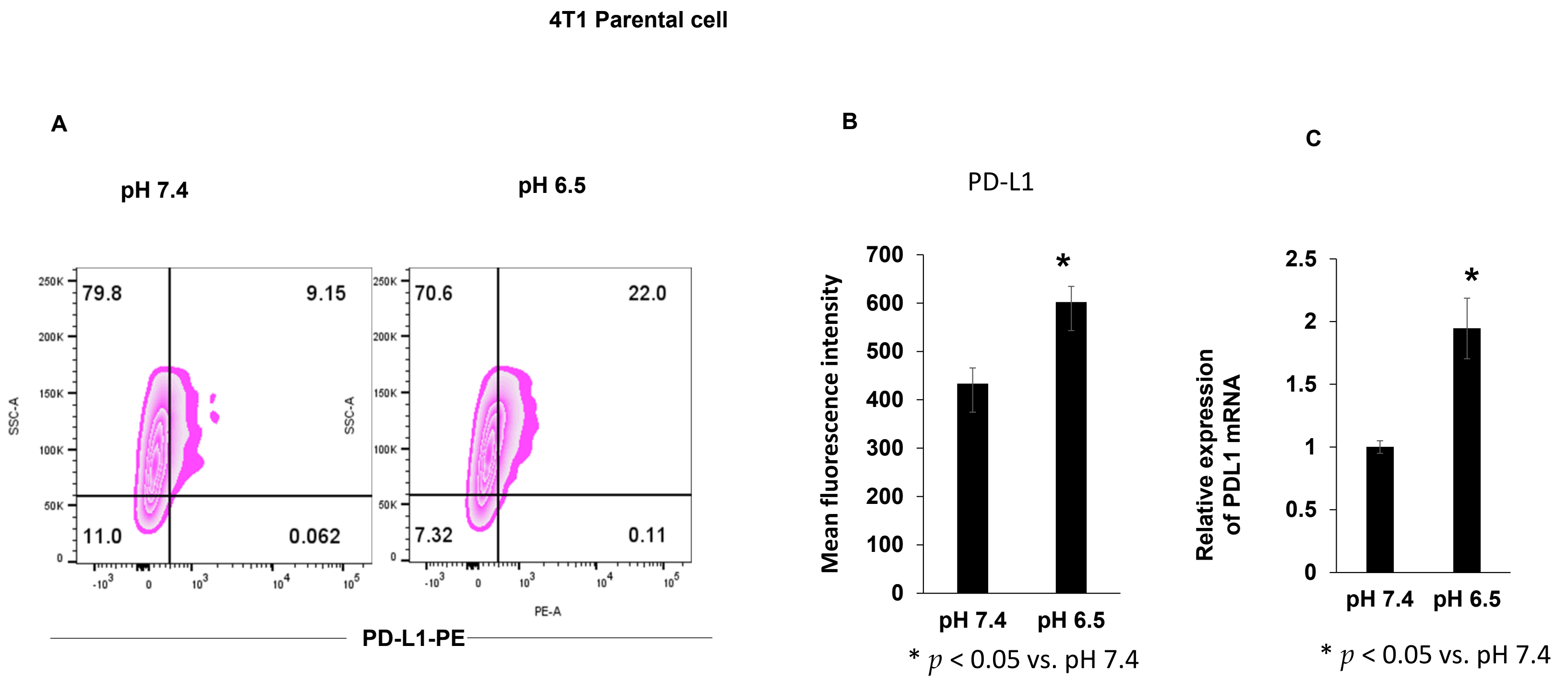

Figure S1. Effect of extracellular acidosis on PD-L1 expression in 4T1 parent cells. The pH of the cell culture medium was adjusted by HCl or NaOH, and 4T1 cells were incubated for 24 h and analyzed by flow cytometry. Flow cytometry dot plots (**A**) and mean fluorescence (**B**) of cell surface PD-L1 expression (* $p < 0.05$ for pH 6.5 vs. pH 7.4).
